# *Mycobacterium bovis* Bacille-Calmette-Guérin Infection Aggravates Atherosclerosis 

**DOI:** 10.3389/fimmu.2020.607957

**Published:** 2020-12-18

**Authors:** Moises A. Huaman, Joseph E. Qualls, Shinsmon Jose, Stephanie M. Schmidt, Anissa Moussa, David G. Kuhel, Eddy Konaniah, Ravi K. Komaravolu, Carl J. Fichtenbaum, George S. Deepe, David Y. Hui

**Affiliations:** ^1^ Division of Infectious Diseases, Department of Internal Medicine, University of Cincinnati College of Medicine, Cincinnati, OH, United States; ^2^ Division of Infectious Diseases, Cincinnati Children’s Hospital Medical Center, Department of Pediatrics, University of Cincinnati College of Medicine, Cincinnati, OH, United States; ^3^ Metabolic Diseases Research Center, Department of Pathology, University of Cincinnati College of Medicine, Cincinnati, OH, United States

**Keywords:** mycobacterium, Bacille-Calmette-Guérin, tuberculosis, atherosclerosis, inflammation, T cells, monocytes

## Abstract

Tuberculosis has been associated with increased risk of atherosclerotic cardiovascular disease. To examine whether mycobacterial infection exacerbates atherosclerosis development in experimental conditions, we infected low-density lipoprotein receptor knockout (*Ldlr* ^-/-^) mice with *Mycobacterium bovis* Bacille-Calmette-Guérin (BCG), an attenuated strain of the *Mycobacterium tuberculosis* complex. Twelve-week old male *Ldlr* ^-/-^ mice were infected with BCG (0.3–3.0x10^6^ colony-forming units) via the intranasal route. Mice were subsequently fed a western-type diet containing 21% fat and 0.2% cholesterol for up to 16 weeks. Age-matched uninfected *Ldlr* ^-/-^ mice fed with an identical diet served as controls. Atherosclerotic lesions in aorta were examined using Oil Red O staining. Changes induced by BCG infection on the immunophenotyping profile of circulating T lymphocytes and monocytes were assessed using flow cytometry. BCG infection increased atherosclerotic lesions in *en face* aorta after 8 weeks (plaque ratio; 0.021±0.01 vs. 0.013±0.01; *p* = 0.011) and 16 weeks (plaque ratio, 0.15±0.13 vs. 0.06±0.02; *p* = 0.003). No significant differences in plasma cholesterol or triglyceride levels were observed between infected and uninfected mice. Compared to uninfected mice, BCG infection increased systemic CD4/CD8 T cell ratio and the proportion of Ly6C^low^ non-classical monocytes at weeks 8 and 16. Aortic plaque ratios correlated with CD4/CD8 T cell ratios (Spearman’s rho = 0.498; *p* = 0.001) and the proportion of Ly6C^low^ non-classical monocytes (Spearman’s rho = 0.629; *p* < 0.001) at week 16. In conclusion, BCG infection expanded the proportion of CD4^+^ T cell and Ly6C^low^ monocytes, and aggravated atherosclerosis formation in the aortas of hyperlipidemic *Ldlr* ^-/-^ mice. Our results indicate that mycobacterial infection is capable of enhancing atherosclerosis development.

## Introduction

It is estimated that a quarter of the world population has latent tuberculosis infection, and about 10 million people develop active tuberculosis each year globally (1, 2). Patients with a history of active tuberculosis have an increased risk of myocardial infarction, ischemic stroke, and peripheral arterial disease, suggesting that mycobacterial infection has a role in atherosclerotic cardiovascular disease (3–6). Although these studies accounted for common traditional cardiovascular risk factors, there is a possibility of residual confounding effects from measured and unmeasured characteristics that may not be fully controlled for in human population-based studies (7). Therefore, there is a need to explore the relationship between mycobacterial infection and atherogenesis in experimental animal models. Furthermore, perturbations in circulating T cell and monocyte subsets have been described in tuberculosis (8, 9). These immune cells play an important role in atherosclerosis development (10), but their correlation with atherosclerotic plaque in the setting of mycobacterial infection is not well characterized.

The pro-atherogenic effects of infectious agents were first described more than 4 decades ago, when Fabricant *et al* reported experimental induction of atherosclerosis by Marek’s virus in chickens (11). Bacterial pathogens including *Chlamydia pneumoniae* (12–14), *Helicobacter pylori* (15), and periodontal organisms such as *Porphyromonas ginvivalis* (16) have been associated with increased atherosclerosis formation in hyperlipidemic animal models. Several potential mechanisms linking infection and atherosclerosis have been described, including direct pathogen invasion of vascular tissue and indirect effects via inflammatory and immune mechanisms (17–19). Previous animal studies using *Mycobacterium bovis* Bacille-Calmette-Guérin (BCG), an attenuated strain of the *Mycobacterium tuberculosis* complex, have shown different modulating effects of infection in atherosclerosis development. In hypercholesterolemic rabbits, subcutaneous *M. bovis* BCG injections enhanced atherogenesis (20). However, a recent study in ApoE*3 Leiden.CETP mice showed reduced atherosclerotic plaque formation after intravenous inoculation of *M. bovis* BCG (21). Notably, the latter study was confounded by a significant reduction of plasma cholesterol levels in the mice infected with *M. bovis* BCG, which likely mediated the final atherosclerosis outcome in this model (22).

To further assess the effects of mycobacteria in atherogenesis, we infected low-density lipoprotein receptor knockout (*Ldlr* ^-/-^) mice with *M. bovis* BCG via the intranasal route. We used this route of infection to mimic the natural respiratory route of acquisition of mycobacterial infections (23). We used *Ldlr* ^-/-^ mice, as *M. bovis* BCG infection was not expected to induce significant plasma cholesterol changes in this atherosclerosis model (24). We aimed at assessing whether *M. bovis* BCG infection exacerbates atherosclerosis development and induces changes on the immunophenotyping profile of circulating T cell and monocytes. We report that *M. bovis* BCG infection expanded the proportion of circulating CD4^+^ T cell and Ly6C^low^ monocytes, and aggravated atherosclerosis formation in murine aorta.

## Materials and Methods

### Mice, Diet, and Study Setting

Twelve-week old male C57BL/6J *Ldlr* ^-/-^ mice were purchased from the Jackson Laboratory and housed at the Laboratory Animal Medical Services (LAMS) within the University of Cincinnati. Mice were anesthetized and inoculated with *M. bovis* BCG [0.3–3.0x10^6^ colony-forming units (CFUs)] via the intranasal route. Mice were subsequently fed a western-type diet (WD) containing 21% fat and 0.2% cholesterol (Envigo TD.88137 diet) for up to 16 weeks. Age-matched uninfected *Ldlr* ^-/-^ mice fed with an identical WD served as controls. Mice were weighed every 2 weeks after initiation of WD to assess for differences in total body weight between groups. The protocols for animal experiments were conducted as per the University of Cincinnati Institutional Animal Care and Use Committee (IACUC) and National Institutes of Health (NIH) guidelines.

### Atherosclerotic Lesion Assessment

Mice were euthanized at 8 and 16 weeks of WD to examine atherosclerotic lesions in aortic root sections and *en face* aorta using Oil Red O staining. Twelve frozen sections per sample were examined throughout the aortic root. Plaque ratios were determined based on plaque area per total area at the aortic root. For *en face* assessments of total aorta, the extent of plaque was measured using the plaque size per aorta area ratio using ImageJ (NIH, Bethesda, MD). Plaque composition was assessed in aortic root sections. We used CD68 antibody (Abcam ab955) at 1:200 dilution and SMC alpha actin (Abcam ab5694) at 1:100 dilution for immunofluorescence staining to assess macrophage and smooth muscle content, respectively. Images were captured using immunofluorescence microscopy (Olympus BX61) and the areas of positive fluorescence per total area of plaque ratios were estimated. Fibrosis was detected by Sirius red staining.

### Circulating Lipids Assessment

Blood was collected via intra-cardiac puncture immediately after euthanasia. Plasma was separated for analysis of circulating lipids. Total plasma cholesterol and triglyceride levels were measured using enzymatic assays (Infinity^TM^ reagents). Lipoprotein distribution was assessed using fast protein liquid chromatography (FPLC).

### Immunophenotyping of Circulating T Cells and Monocytes

T cell and monocyte subsets were assessed using flow cytometry. After two rounds of red blood cell lysis, FcγII/III receptors were blocked using anti-mouse CD32/CD16 for 15 min at 4°C (Leinco clone YT1.24). Cells were stained using antibodies for 30 min at 4°C at 1:100 dilution in FACS buffer (buffered salt solution with 0.5% bovine serum albumin; Leinco). The T cell panel included the following antibodies and conjugated fluorochromes: CD45.2 (clone 104)/brilliant violet 711 and CD3e (clone 145-2C11)/PerCP-Cy5.5 from BD Biosciences; CD4 (GK1.5)/FITC, CD8b (H35-17.2)/PE-Cy7, CD44 (IM7)/Brilliant Violet 421, CD25 (PC61.5)/APC, Foxp3 (clone NRRF-30)/PE from eBioscience. The monocyte panel included CD45.2 (clone 104)/brilliant violet 711, CD11b (clone M1/90)/APC-Cy7, CD11c (clone HL3)/PE-Cy7, CD115 (clone T38-320)/PE, Ly6G (clone 1A8)/PerCP-Cy5.5, and Ly6C (clone AL-21)/APC from BD Biosciences. Cells stained with the T cell panel antibodies were washed and fixed with Fix/Perm buffer and then stained for intracellular FoxP3 (eBioscience clone NRRF-30/PE). Cells stained with the monocyte panel antibodies were washed and fixed with Fix/Perm buffer and then stained for intracellular NOD2 (Novus Biologicals clone 2D9/Alexa Fluor 488). Flow cytometric data were acquired using a BD^TM^ LSR II. Data were analyzed using FlowJo v10 software. We used Fluorescence minus one (FMO) controls to set our gates.

### Mycobacteria CFU Enumeration

Right lungs and spleens were harvested for CFU enumeration. Lung and spleen tissues were homogenized in 5 ml of sterile phosphate-buffered saline (PBS) and serially diluted on 7H10 agar (262710, BD Diagnostic) supplemented with 2.5 mg/L amphotericin B (A9528, Sigma), 26 mg/L polymyxin B sulfate (P4932-5MU, Sigma), 20 mg/L trimethoprim lactate (T0667-260mg, Sigma), 50 mg/L carbenicillin disodium (C3416-1G, Sigma), and OADC enrichment (R450605, Fischer) (25). CFUs were quantified following humidified incubation at 37°C for 2 to 3 weeks.

### Statistical Analyses

We used unpaired Student’s t-test for group comparisons of numeric variables and flow cytometry data. To assess the correlation between immune parameters and plaque ratio, we used the Spearman’s correlation test. Analyses were carried out in Stata v12 (College Station, TX); *p* values <0.05 were considered statistically significant. All *p* values were 2-tailed.

## Results

To confirm that inoculation with *M. bovis* BCG via the intranasal route induced a persistent mycobacterial infection in our murine model, we cultured lung and spleen homogenates of mice at time of euthanasia. Mean *M. bovis* BCG CFU in the infected group was 3.6x10^6^±1.6x10^6^ CFU/g in lung and 1.1x10^6^±6.5x10^5^ CFU/g in spleen at 8 weeks post challenge. The *M. bovis* BCG CFU remained detectable at 5.2x10^5^±4.9x10^5^ CFU/g in lung and 1.1x10^6^±9x10^5^ CFU/g in spleen by 16 weeks. Mycobacterial colonies were not detectable from the lungs or spleens of uninfected control mice. These findings confirmed that we were able to establish persistent mycobacterial infection in our experimental model.

### *M. bovis* BCG Increases Atherosclerosis in *En Face Aorta*

**Figure 1:**
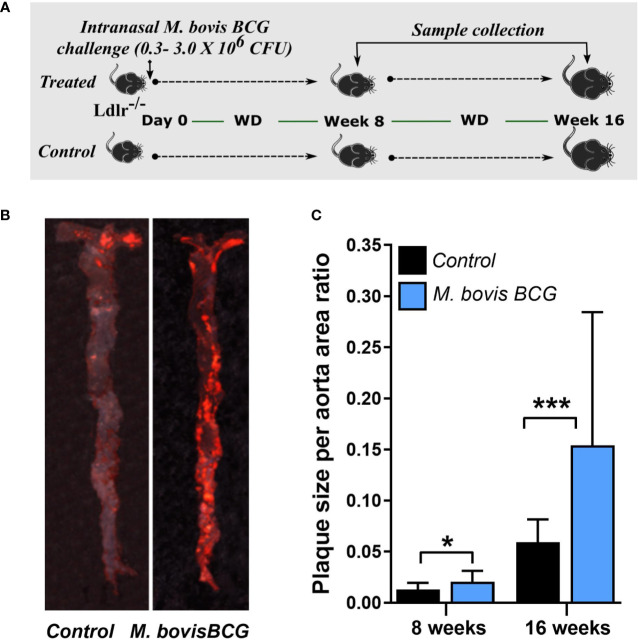
*M. bovis* BCG increases *en face* aorta atherosclerosis by 8 and 16 weeks post-challenge. **(A)** Twelve-week old male *Ldlr^-/-^* mice were inoculated with *M. bovis* BCG (0.3–3.0x10^6^ CFU) via the intranasal route. Mice were fed a western-type diet for up to 16 weeks. Age-matched uninfected *Ldlr* ^-/-^ mice fed with an identical diet served as controls. **(B)** Atherosclerotic lesions in *en face* aorta were examined using Oil Red O staining at weeks 8 and 16. Data are representative of the aortae of one *M. bovis* BCG-infected mouse and one control mouse at 16 weeks. **(C)** Plaque burden was quantified by the plaque size per aorta ratio in *M. bovis* BCG-infected (blue) and control mice (black). Data are mean ± SD. n = 20 mice per group pooled from 2 independent experiments. Significance was determined by Student’s *t*-test. *p < 0.05; ***p < 0.001.

**Figure 2:**
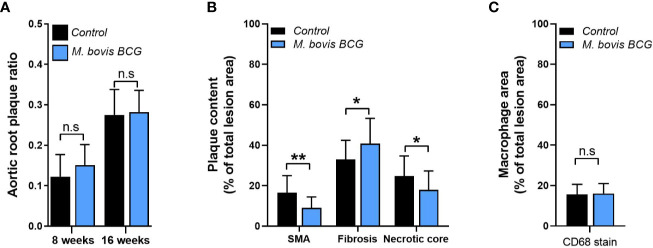
*M. bovis* BCG does not increase extent of aortic root atherosclerosis. *Ldlr^-/-^* mice were inoculated with *M. bovis* BCG (0.3–3.0x10^6^ CFU) via the intranasal route. Mice were fed a western-type diet for 8 to 16 weeks. Age-matched uninfected *Ldlr* ^-/-^ mice fed with an identical diet served as controls. **(A)** Plaque area per total area of aortic root ratios were quantified in *M. bovis* BCG-infected (blue) and control mice (black) at weeks 8 and 16. **(B)** Aortic root plaque composition of smooth muscle (using smooth muscle alpha actin staining; SMA), fibrosis content (using Syrius red staining), and necrotic core were quantified as percentage per total lesion area at week 16. **(C)** Macrophage content using CD68 staining was quantified as percentage per total lesion area at week 16. Data are mean ± SD. n = 20 mice per group pooled from 2 independent experiments. Significance was determined by Student’s *t*-test. *p < 0.05; **p < 0.01; n.s., non-significant (*p* > 0.05).

*M. bovis* BCG infection significantly increased atherosclerotic lesions in *en face* aorta after 8 weeks (plaque ratio; 0.021 ± 0.01 vs. 0.013 ± 0.01; *p* = 0.011; **Figures 1A, C**) and 16 weeks of inoculation (plaque ratio, 0.15 ± 0.13 vs. 0.06 ± 0.02; *p* = 0.003; **Figures 1B, C**). The aortic root sections showed similar plaque involvement in infected and uninfected mice at week 8 (plaque ratio, 0.15 ± 0.06 vs. 0.13 ± 0.07; *p* = 0.402; **Figure 2A**) and week 16 (plaque ratio; 0.28 ± 0.05 vs. 0.28 ± 0.06; *p* = 0.726; **Figure 2A**). Plaque composition analysis of aortic root sections revealed decreased smooth muscle-specific alpha-actin expression (0.09 ± 0.05 vs. 0.17 ± 0.08; *p* = 0.002; **Figure 2B**), decreased necrotic core content (0.18 ± 0.09 vs. 0.25 ± 0.09; *p* = 0.033; **Figure 2B**), and increased fibrosis (0.41 ± 0.12 vs. 0.33 ± 0.09; *p* = 0.034; **Figure 2B**) among *M. bovis* BCG-infected mice compared to uninfected mice. Similar content of CD68^+^ macrophages was observed in aortic root sections of *M. bovis* BCG-infected and uninfected mice (0.161 ± 0.05 vs. 0.157 ± 0.05; *p* = 0.779; **Figure 2C**). We were unable to detect mycobacteria in atherosclerotic lesions by acid fast bacilli staining or immunofluorescence staining using anti-mycobacterium Ag85B antibodies (**Supplementary Figure 1**). Overall, our experiments showed that *M. bovis* BCG exacerbated the extent of atherosclerosis within the aorta. However, the effect of mycobacteria in atherosclerosis was not noticeable in the aortic root, perhaps because this area of high turbulence is already prone to plaque formation in the setting of high fat diet and hyperlipidemia (26), regardless of infection.

### *M. bovis* BCG Infection Does Not Induce Significant Changes in Body Weight or Circulating Lipids in Ldlr^-/-^ Mice

Mice infected with *M. bovis* BCG displayed no differences in total body weight through the course of 16 weeks of WD, compared to uninfected control mice (baseline body weight, 24.2 ± 1.5 vs. 23.8 ± 1.5, *p* = 0.552; body weight at 8 weeks of WD, 34.4 ± 3.6 vs. 36 ± 5.1, *p* = 0.287; body weight at 16 weeks of WD, 35.2 ± 3.2 vs. 33.8 ± 2.5, *p* = 0.292; **Supplementary Figure 2A**). As expected in this model and diet conditions, mice were overall hyperlipidemic, but there were no significant differences in plasma cholesterol (1,160 ± 230 mg/dL vs. 1,278 ± 298 mg/dL; *p* = 0.359; **Supplementary Figure 2B**) or triglycerides (340 ± 125 mg/dL vs. 413 ± 154 mg/dL; *p* = 0.284; **Supplementary Figure 2C**) between infected vs. uninfected mice at 16 weeks. FPLC chromatograms showed a similar distribution of triglyceride and cholesterol fractions at week 16 (**Supplementary Figures 2D, E**). Similarly, no significant differences in plasma cholesterol (887 ± 444 vs. 1073 ± 353; *p* = 0.151; **Supplementary Figure 2B**) or triglycerides (256 ± 138 vs. 319 ± 98; *p* = 0.101; **Supplementary Figure 2C**) were observed between infected vs. uninfected mice at week 8. These results indicated that the aggravated atherosclerosis findings associated with *M. bovis* BCG were not related to increased amounts of circulating lipids induced by infection.

### *M. bovis* BCG Infection Induces an Expansion of CD4^+^ T Cells and Monocytes

**Figure 3:**
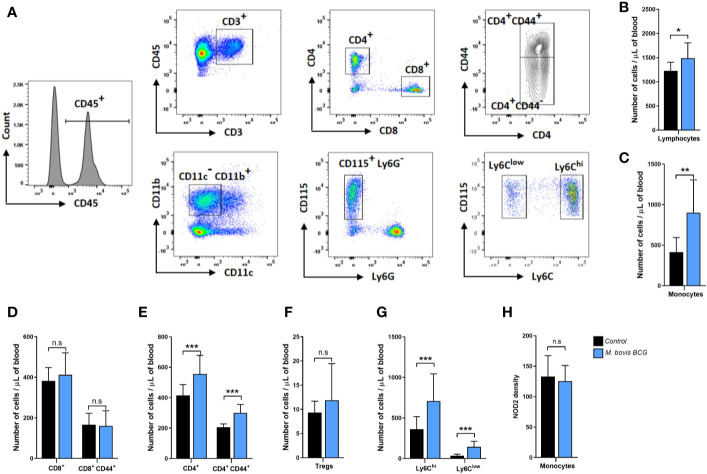
*M. bovis* BCG induces CD4^+^ T cell and monocyte activation by week 8. Flow cytometry analysis of blood lymphocytes and monocytes from *M. bovis* BCG-infected and control mice at 8 weeks. *Ldlr^-/-^* mice were inoculated with *M. bovis* BCG (0.3–3.0x10^6^ CFU) via the intranasal route. Mice were fed a western-type diet for 8 weeks. Age-matched uninfected *Ldlr* ^-/-^ mice fed with an identical diet served as controls. **(A)** Representative flow cytometry plots showing the gating strategy used for identifying subsets of T cells and monocytes. T cells (upper panel) were defined as CD45^+^ CD3^+^ cells and seperated into CD4^+^ and CD8^+^ T cell subsets. Expression of CD44 was further assessed within T cell subsets. Monocytes (lower panel) were defined as CD45^+^ CD3^-^ CD11b^+^ CD11c^-^ CD115^+^ Ly6G^-^ cells. Monocyte subsets were defined based on Ly6C expression. **(B–H)** Number of lymphocytes **(B)**, monocytes **(C)**, T cell subsets **(D–F)**, monocyte subsets **(G)** and monocyte NOD2 MFI **(H)** from *M. bovis* BCG-infected (blue) and control mice (black). Data are mean ± SD. n = 10 mice per group. Significance was determined by Student’s *t*-test. **p < 0.01; ***p < 0.001; n.s., non-significant (*p* > 0.05).

**Figures 3A–H** summarize the flow cytometry gating strategy and key immunophenotyping findings of circulating T cells and monocytes in *M. bovis* BCG-infected and control mice. *M. bovis* BCG infection induced an increase in circulating T lymphocytes (1,490 ± 316 cells/ul vs. 1,227 ± 177 cells/ul; *p* = 0.034; **Figure 3B**) and monocytes (901 ± 405 cells/ul vs. 414 ± 180 cells/ul; *p* < 0.003; **Figure 3C**) by 8 weeks post-infection. When we assessed T lymphocyte subsets, *M. bovis* BCG infection increased total CD4^+^ T cell counts (556 ± 122 cells/ul vs. 416 ± 71 cells/ul; *p* < 0.005; **Figure 3E**) but not CD8^+^ T cells (413 ± 109 cells/ul vs. 382 ± 66 cells/ul; *p* = 0.468; **Figure 3D**). There was an increased number of CD4^+^ T cells expressing the activation marker CD44 in BCG-infected vs. uninfected mice (211 ± 102 cells/ul vs. 150 ± 61 cell/ul; *p* < 0.027; **Figure 3E**). CD4^+^ FoxP3^+^ T cells were similar between groups (12 ± 8 cells/ul vs. 9 ± 2 cells/ul; *p* = 0.155; **Figure 3F**). When we assessed monocyte subsets, *M. bovis* BCG infection increased the numbers of Ly6C^high^ (709 ± 335 cells/ul vs. 362 ± 155 cells/ul; *p* < 0.008; **Figure 3G**) and Ly6C^low^ (145 ± 68 cells/ul vs. 35 ± 19 cells/ul; *p* < 0.001; **Figure 3G**) monocytes at week 8. NOD2 receptor density was similar in total monocytes and monocyte subsets of BCG-infected and uninfected mice (mean fluorescence density in total monocytes, 125 ± 26 vs. 133 ± 34; *p* = 0.577; **Figure 3H**).

### The CD4/CD8 T Cell Ratio and the Proportion of Ly6C^low^ Monocytes Correlate With the Extent of Aortic Plaque

**Figure 4:**
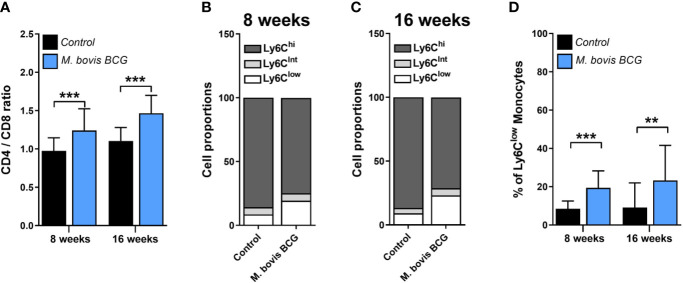
*M. bovis* BCG increases the CD4/C8 ratio and the proportion of Ly6C^low^ monocytes. *Ldlr^-/-^* mice were inoculated with *M. bovis* BCG (0.3–3.0x10^6^ CFU) via the intranasal route. Mice were fed a western-type diet for 8 to 16 weeks. Age-matched uninfected *Ldlr* ^-/-^ mice fed with an identical diet served as controls. **(A)** CD4/CD8 T cell ratios from *M. bovis* BCG and control mice at weeks 8 and 16. **(B, C)** Mean proportions of Ly6C^low^, Ly6C^intermediate^ and Ly6C^high^ monocyte subsets in blood on **(B)** week 8 and **(C)** week 16. **(D)** Percentage of Ly6C^low^ monocytes in blood. Data are means ± SD for A, D. Mean for B, C. n = 10 mice per group; pooled from 2 independent experiments. Significance was determined by Student’s *t*-test. **p < 0.01; ***p < 0.001.

**Figure 5:**
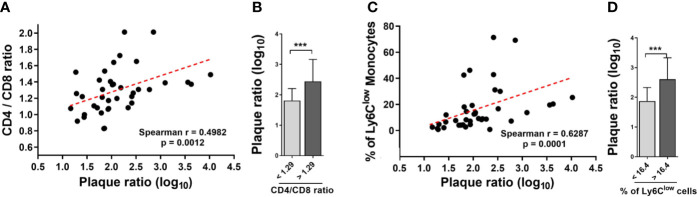
The CD4/CD8 T cell ratio and the proportion of Ly6C^low^ monocytes correlate with the extent of aortic plaque by week 16. *Ldlr^-/-^* mice were inoculated with *M. bovis* BCG (0.3–3.0x10^6^ CFU) via the intranasal route. Mice were fed a western-type diet for 16 weeks. Age-matched uninfected *Ldlr* ^-/-^ mice fed with an identical diet served as controls. **(A)** Spearman correlation of blood CD4/CD8 T cell ratios with aorta plaque ratio. **(B)** Aorta plaque ratio in mice grouped as below or above CD4/CD8 ratio mean. **(C)** Spearman correlation of the proportion of Ly6C^low^ monocytes and aorta plaque ratio. **(D)** Aorta plaque ratio in mice grouped as below or above mean proportion of Ly6C^low^ monocytes in blood. n = 40; data are pooled from 2 independent experiments. Significance was determined by Spearman’s correlation test for **(A**, **C)**. Significance was determined using Student’s *t*-test for **(B**, **D)**. ***p < 0.001.

*M. bovis* BCG infection induced an increase in the CD4/CD8 ratio (1.24 ± 0.17 vs. 0.97 ± 0.28 at week 8; *p* < 0.001; 1.47 ± 0.18 vs. 1.11 ± 0.23 at week 16; *p* < 0.001; **Figure 4A**). In addition, we observed that *M. bovis* BCG infection led to an increase in the proportion of Ly6C^low^ non-classical monocytes (19 vs. 9% at week 8; *p* < 0.001; 23 vs. 9% at week 16; *p* = 0.009; **Figures 4B–D**), compared to uninfected mice. CD4/CD8 ratio (Spearman’s rho = 0.498; *p* = 0.001; **Figures 5A–B**) and the proportion of Ly6C^low^ monocytes (Spearman’s rho = 0.629; *p* < 0.001; **Figures 5C–D**) correlated with aorta plaque formation at week 16. None of the other immunophenotyping parameters studied correlated with aortic plaque.

## Discussion

*M. bovis* BCG infection increased the extent of atherosclerosis formation in the aortas of WD-fed hyperlipidemic *Ldlr* ^-/-^ mice. Circulating lipid levels were not significantly increased in BCG-infected mice compared to uninfected mice, and therefore do not explain the observed differences in atherosclerosis. Compared to uninfected controls, *M. bovis* BCG-infected mice exhibited increased CD4^+^ T cell and monocyte driven systemic immune activation. Overall, our results indicate that mycobacterial infection is capable of enhancing atherosclerosis development.

In a prior study of rabbits inoculated with two subcutaneous injections of *M. bovis* BCG and fed with cholesterol-supplemented diet, infected rabbits displayed increased atherosclerotic lesions in thoracic aorta compared to uninfected controls with similar plasma cholesterol levels (20). However, a recent study in APOE*3-Leiden.CETP mice showed that intravenous inoculation of *M. bovis* BCG was associated with decreased atherosclerosis formation in the aortic root after 6 weeks of infection (21). In the latter model, infection induced lower plasma cholesterol levels compared to uninfected controls, which may have affected the atherosclerosis outcome of the experiments. Our data shows that *M. bovis* BCG infection is capable of increasing atherosclerosis formation in aorta, under similar hypercholesterolemia conditions. Furthermore, we show for the first time that inoculation with *M. bovis* BCG via the respiratory route (which is the most common route of acquisition of mycobacterial infection in humans) exacerbated atherosclerosis and thus supports a pathogenic role of mycobacterial infection in plaque formation.

Population-based studies have indicated an increased risk of atherosclerotic cardiovascular disease in persons with a history of tuberculosis disease (3–6). In addition, recent studies have shown that latent tuberculosis infection is associated with higher rates of coronary artery stenosis and spontaneous acute myocardial infarction (27, 28). These data in humans suggest that the interplay between mycobacteria and cardiovascular disease can occur at different stages of mycobacterial infection. We were able to recover viable mycobacteria from lung and spleen tissues after 16 weeks of *M. bovis* BCG inoculation, suggesting that our model may be more representative of conditions where there is mycobacterial persistence. This is true for active tuberculosis disease, but may also occur within the spectrum of subclinical tuberculosis and latent tuberculosis infection, as the “latent” state encompasses a wide range of host-pathogen interactions (29), some of which may result in residual bacterial replication and/or enhanced systemic immune activation (30–32).

We found that *M. bovis* BCG infection induced monocyte and CD4^+^ T cell driven systemic immune activation, which is in line with results from prior studies of host immune responses to mycobacteria (20, 33). The contribution of these immune cells in atherosclerosis development has been well characterized (34), and likely provides a mechanistic link between infection and atherogenesis. Both the CD4/CD8 T cell ratio and the proportion of Ly6C^low^ monocyte were associated with plaque burden in our study. However, a limitation of our study is that we did not conduct in-depth mechanistic experiments to assess the role of specific immune or mycobacterial parameters in atherogenesis. Future studies detailing specific tissue dissemination—including interactions of *M. bovis* BCG, inflammatory cells, and other stromal cells within atherosclerotic plaque—are needed. Whether targeted immune mechanisms mediate the effects of mycobacterial infection in atherosclerosis can be assessed in future studies. Of note, an increased systemic CD4/CD8 T cell ratio was recently found to be strongly and independently associated with coronary artery disease in elderly individuals (35). In human atherosclerotic plaque, there is a progressive expansion of the CD4^+^ T cell compartment as atherosclerotic lesions evolve (36). Although a decreased systemic CD4/CD8 T cell ratio has also been associated with increased human atherosclerosis, this phenomenon has been observed primarily in persons living with HIV/AIDS (37).

*M. bovis BCG* infection increased the number of circulating monocytes in our model. Monocytosis has been observed in human and murine myocardial infarction (38). Furthermore, a recent study demonstrated that monocyte recruitment from the circulation into aortic plaque is required for atherosclerosis progression (39). When monocyte subsets were analyzed, *M. bovis* BCG-infected mice showed a higher proportion of non-classical Ly6C^low^ circulating monocytes, which may be in response to increased endothelial injury, and could drive increased fibrosis in vascular tissue (40). Alternatively, our results may indicate a pro-atherogenic contribution of non-classical Ly6C^low^ monocytes in atherogenesis. Despite Ly6C^low^ monocytes being known to promote endothelial repair (41), a recent study indicated that these cells are involved in early plaque development (42). Furthermore, experimental inhibition of CCR5, a chemokine receptor preferentially involved in Ly6C^low^ monocyte recruitment to atherosclerotic plaque (43), has resulted in decreased atherosclerosis formation (42, 44). Of note, triggering of the NOD2 receptor has been reported to promote conversion of Ly6C^high^ into Ly6C^low^ monocytes with patrolling properties (45). We did not see significant differences in NOD2 receptor expression between BCG-infected and uninfected mice; however, these results do not exclude the possibility of differential downstream NOD2 signaling. Monocytes exposed to *M. bovis* BCG develop a prolonged pro-inflammatory phenotype via epigenetic changes in histone methylation at the level of bone marrow progenitors, a phenomenon coined as trained immunity (46). In addition to central trained immunity, peripheral trained immunity of blood monocytes and tissue macrophages has also been described (47, 48). Trained monocyte-derived macrophages have an augmented production of pro-atherogenic cytokines including IL-1β, IL-6, and tumor necrosis factor-α, and are more prone to foam cell formation upon exposure to a second non-specific stimulus (49, 50). Thus, trained immunity is gaining recognition as a plausible mechanistic link between infection and atherosclerosis development (22, 51). Furthermore, classical pro-atherogenic stimuli such as oxidized-LDL also induce a trained immune phenotype in monocytes (50), suggesting that both infectious and non-infectious triggers might contribute to atherosclerosis through shared disease pathways. Future studies assessing trained immunity and epigenetic reprogramming of Ly6C^low^ and Ly6C^high^ monocytes and their effects in atherosclerotic plaque may provide insights into mechanisms of atherosclerosis in the setting of mycobacterial infection.

In conclusion, *M. bovis* BCG infection increased the extent of atherosclerosis development in the aortas of WD-fed hyperlipidemic *Ldlr* ^-/-^ mice. Our results indicate that mycobacterial infection is capable of enhancing atherosclerosis development, and provide experimental evidence for previously reported links between tuberculosis and atherosclerotic cardiovascular disease in humans.

## Data Availability Statement

The primary data supporting the conclusions of this article will be made available by the authors, upon reasonable request.

## Ethics Statement

The animal study was reviewed and approved by University of Cincinnati Institutional Animal Care and Use Committee (IACUC).

## Author Contributions

MH, JQ, CF, GD, and DH contributed to the conception and design of the study. MH, JQ, SJ, SS, AM, DK, EK, and RK conducted the investigation and experiments. MH, SJ, DK, and EK performed statistical analyses. All authors interpreted data. MH wrote the first draft of the report. All authors contributed to the article and approved the submitted version.

## Funding

This work was supported by the National Center for Advancing Translational Science (grant number KL2 TR001426 to MH), the National Institute of Allergy and Infectious Diseases (grant number R01 AI116668 to JQ), and the National Institute of Diabetes and Digestive and Kidney Diseases (grant number R01 DK074932 to DH) at the National Institutes of Health. MH also received support from the Department of Internal Medicine at the University of Cincinnati College of Medicine. The contents are solely the responsibility of the authors and do not necessarily represent the official views of the National Institutes of Health or the institutions with which the authors are affiliated. The funding source had no role in the study design; in the collection, analysis, and interpretation of data; in the writing of the report; or in the decision to submit the report for publication.

## Conflict of Interest

CF has received research support to the University of Cincinnati from Gilead, Pfizer, BMS, ViiV, Janssen, and Merck.

The remaining authors declare that the research was conducted in the absence of any commercial or financial relationships that could be construed as a potential conflict of interest.

## Supplementary Material

Supplementary Figure 1 Detection of mycobacteria in atherosclerotic lesions. Frozen sections of aortic roots isolated from *M. bovis* BCG-infected and uninfected *Ldlr^-/-^* mice were histologically stained with acid fast reagents by placing in Carbol-fuchsin solution (Sigma-Aldrich, catalog No. HT801) for 1 min followed by 2 min of counterstain with Malachite Green solution (Sigma-Aldrich, catalog No. HT802). The frozen sections were also subjected to immunofluorescence staining with the anti-*Mycobacterium tuberculosis* antibodies Ag85B (Abcam, catalog No. ab43019) with DAPI counterstain to identify the nucleus. The scale bars represent 100 µm. Note that no mycobacteria were detectable in the atherosclerotic lesions from either *M. bovis* BCG infected or uninfected mice.

Supplementary Figure 2
*M. bovis* BCG infection does not induce significant changes in body weight or circulating lipids in *Ldlr* ^-/-^ mice. *Ldlr^-/-^* mice were inoculated with *M. bovis* BCG (0.3–3.0x10^6^ CFU) via the intranasal route. Mice were fed a western-type diet for 8 to 16 weeks. Age-matched uninfected *Ldlr* ^-/-^ mice fed with an identical diet served as controls. **(A)** Body weight at baseline, 8 weeks, and 16 weeks of western-type diet in *M.bovis* BCG infected (blue) and control mice (black). **(B)** Plasma cholesterol levels in mg/dL at weeks 8 and 16. **(C)** Plasma triglyceride levels in mg/dL at weeks 8 and 16. Data are means ± SD. n = 20 mice per group; pooled from 2 independent experiments. Significance was determined by Student’s t-test. n.s., non-significant (*p* > 0.05). Fast protein liquid chromatography (FPLC) profiles of total cholesterol **(D)** and tryglicerides **(E)** in *M.bovis* BCG infected and control mice at week 16.

## References

[1] World Health Organization Global tuberculosis report 2018. Geneva, Switzerland: World Health Organization (2018).

[2] Houben RM Dodd PJ The Global Burden of Latent Tuberculosis Infection: A Re-estimation Using Mathematical Modelling. PloS Med (2016) 13(10):e1002152. 10.1371/journal.pmed.1002152 27780211 PMC5079585

[3] Sheu JJ Chiou HY Kang JH Chen YH Lin HC Tuberculosis and the risk of ischemic stroke: a 3-year follow-up study. Stroke (2010) 41(2):244–9. 10.1161/STROKEAHA.109.567735 20035070

[4] Chung WS Lin CL Hung CT Chu YH Sung FC Kao CH Tuberculosis increases the subsequent risk of acute coronary syndrome: a nationwide population-based cohort study. Int J Tuberc Lung Dis (2014) 18(1):79–83. 10.5588/ijtld.13.0288 24365557

[5] Huaman MA Kryscio RJ Fichtenbaum CJ Henson D Salt E Sterling TR Tuberculosis and risk of acute myocardial infarction: a propensity score-matched analysis. Epidemiol Infect (2017) 145(7):1363–7. 10.1017/S0950268817000279 PMC5616129 28202093

[6] Wang SH Chien WC Chung CH Lin FH Peng CK Chian CF Tuberculosis increases the risk of peripheral arterial disease: A nationwide population-based study. Respirology (2017) 22(8):1670–6. 10.1111/resp.13117 28681508

[7] Basham CA Smith SJ Romanowski K Johnston JC Cardiovascular morbidity and mortality among persons diagnosed with tuberculosis: A systematic review and meta-analysis. PloS One (2020) 15(7):e0235821. 10.1371/journal.pone.0235821 32649721 PMC7351210

[8] Sampath P Moideen K Ranganathan UD Bethunaickan R Monocyte Subsets: Phenotypes and Function in Tuberculosis Infection. Front Immunol (2018) 9:1726. 10.3389/fimmu.2018.01726 30105020 PMC6077267

[9] Barcelos W Martins-Filho OA Guimaraes TM Oliveira MH Spindola-de-Miranda S Carvalho BN Peripheral blood mononuclear cells immunophenotyping in pulmonary tuberculosis patients before and after treatment. Microbiol Immunol (2006) 50(8):597–605. 10.1111/j.1348-0421.2006.tb03834.x 16924144

[10] Hansson GK Hermansson A The immune system in atherosclerosis. Nat Immunol (2011) 12(3):204–12. 10.1038/ni.2001 21321594

[11] Fabricant CG Fabricant J Litrenta MM Minick CR Virus-induced atherosclerosis. J Exp Med (1978) 148(1):335–40. 10.1084/jem.148.1.335 PMC2184908 209124

[12] Moazed TC Campbell LA Rosenfeld ME Grayston JT Kuo CC Chlamydia pneumoniae infection accelerates the progression of atherosclerosis in apolipoprotein E-deficient mice. J Infect Dis (1999) 180(1):238–41. 10.1086/314855 10353889

[13] Blessing E Campbell LA Rosenfeld ME Chough N Kuo CC Chlamydia pneumoniae infection accelerates hyperlipidemia induced atherosclerotic lesion development in C57BL/6J mice. Atherosclerosis (2001) 158(1):13–7. 10.1016/S0021-9150(00)00758-9 11500169

[14] Chen S Shimada K Zhang W Huang G Crother TR Arditi M IL-17A is proatherogenic in high-fat diet-induced and Chlamydia pneumoniae infection-accelerated atherosclerosis in mice. J Immunol (2010) 185(9):5619–27. 10.4049/jimmunol.1001879 PMC3046880 20935201

[15] Yang S Xia YP Luo XY Chen SL Li BW Ye ZM Exosomal CagA derived from Helicobacter pylori-infected gastric epithelial cells induces macrophage foam cell formation and promotes atherosclerosis. J Mol Cell Cardiol (2019) 135:40–51. 10.1016/j.yjmcc.2019.07.011 31352044

[16] Hayashi C Viereck J Hua N Phinikaridou A Madrigal AG Gibson FC,3 Porphyromonas gingivalis accelerates inflammatory atherosclerosis in the innominate artery of ApoE deficient mice. Atherosclerosis (2011) 215(1):52–9. 10.1016/j.atherosclerosis.2010.12.009 PMC3057233 21251656

[17] Campbell LA Rosenfeld ME Infection and Atherosclerosis Development. Arch Med Res (2015) 46(5):339–50. 10.1016/j.arcmed.2015.05.006 PMC4524506 26004263

[18] Epstein SE Zhu J Najafi AH Burnett MS Insights into the role of infection in atherogenesis and in plaque rupture. Circulation (2009) 119(24):3133–41. 10.1161/CIRCULATIONAHA.109.849455 19546396

[19] Libby P Loscalzo J Ridker PM Farkouh ME Hsue PY Fuster V Inflammation, Immunity, and Infection in Atherothrombosis: JACC Review Topic of the Week. J Am Coll Cardiol (2018) 72(17):2071–81. 10.1016/j.jacc.2018.08.1043 PMC6196735 30336831

[20] Lamb DJ Eales LJ Ferns GA Immunization with bacillus Calmette-Guerin vaccine increases aortic atherosclerosis in the cholesterol-fed rabbit. Atherosclerosis (1999) 143(1):105–13. 10.1016/S0021-9150(98)00284-6 10208485

[21] van Dam AD Bekkering S Crasborn M van Beek L van den Berg SM Vrieling F BCG lowers plasma cholesterol levels and delays atherosclerotic lesion progression in mice. Atherosclerosis (2016) 251:6–14. 10.1016/j.atherosclerosis.2016.05.031 27232458

[22] Leentjens J Bekkering S Joosten LAB Netea MG Burgner DP Riksen NP Trained Innate Immunity as a Novel Mechanism Linking Infection and the Development of Atherosclerosis. Circ Res (2018) 122(5):664–9. 10.1161/CIRCRESAHA.117.312465 29367213

[23] Small PM Fujiwara PI Management of tuberculosis in the United States. N Engl J Med (2001) 345(3):189–200. 10.1056/NEJM200107193450307 11463015

[24] Ovchinnikova OA Berge N Kang C Urien C Ketelhuth DF Pottier J Mycobacterium bovis BCG killed by extended freeze-drying induces an immunoregulatory profile and protects against atherosclerosis. J Intern Med (2014) 275(1):49–58. 10.1111/joim.12127 23962000

[25] Lange SM McKell MC Schmidt SM Zhao J Crowther RR Green LC l-Arginine Synthesis from l-Citrulline in Myeloid Cells Drives Host Defense against Mycobacteria In Vivo. J Immunol (2019) 202(6):1747–54. 10.4049/jimmunol.1801569 PMC6401247 30710047

[26] VanderLaan PA Reardon CA Getz GS Site specificity of atherosclerosis: site-selective responses to atherosclerotic modulators. Arterioscler Thromb Vasc Biol (2004) 24(1):12–22. 10.1161/01.ATV.0000105054.43931.f0 14604830

[27] Alsayed Hasanain AF El-Maghraby KM AA AN AM SM A-A Bakkar SM Latent tuberculosis infection among patients with coronary artery stenosis: A case-Control study. Int J Mycobacteriol (2018) 7(2):143–7. 10.4103/ijmy.ijmy_34_18 29900890

[28] Huaman MA Ticona E Miranda G Kryscio RJ Mugruza R Aranda E The Relationship Between Latent Tuberculosis Infection and Acute Myocardial Infarction. Clin Infect Dis (2018) 66(6):886–92. 10.1093/cid/cix910 PMC5850031 29069328

[29] Barry CE,3 Boshoff HI Dartois V Dick T Ehrt S Flynn J The spectrum of latent tuberculosis: rethinking the biology and intervention strategies. Nat Rev Microbiol (2009) 7(12):845–55. 10.1038/nrmicro2236 PMC4144869 19855401

[30] Gill WP Harik NS Whiddon MR Liao RP Mittler JE Sherman DR A replication clock for Mycobacterium tuberculosis. Nat Med (2009) 15(2):211–4. 10.1038/nm.1915 PMC2779834 19182798

[31] Gideon HP Phuah J Myers AJ Bryson BD Rodgers MA Coleman MT Variability in tuberculosis granuloma T cell responses exists, but a balance of pro- and anti-inflammatory cytokines is associated with sterilization. PloS Pathog (2015) 11(1):e1004603. 10.1371/journal.ppat.1004603 25611466 PMC4303275

[32] Huaman MA Henson D Rondan PL Ticona E Miranda G Kryscio RJ Latent tuberculosis infection is associated with increased unstimulated levels of interferon-gamma in Lima, Peru. PloS One (2018) 13(9):e0202191. 10.1371/journal.pone.0202191 30212453 PMC6136705

[33] Philips JA Ernst JD Tuberculosis pathogenesis and immunity. Annu Rev Pathol (2012) 7:353–84. 10.1146/annurev-pathol-011811-132458 22054143

[34] Tabas I Lichtman AH Monocyte-Macrophages and T Cells in Atherosclerosis. Immunity (2017) 47(4):621–34. 10.1016/j.immuni.2017.09.008 PMC5747297 29045897

[35] Gao P Rong HH Lu T Tang G Si LY Lederer JA The CD4/CD8 ratio is associated with coronary artery disease (CAD) in elderly Chinese patients. Int Immunopharmacol (2017) 42:39–43. 10.1016/j.intimp.2016.11.007 27866071

[36] van Dijk RA Duinisveld AJ Schaapherder AF Mulder-Stapel A Hamming JF Kuiper J A change in inflammatory footprint precedes plaque instability: a systematic evaluation of cellular aspects of the adaptive immune response in human atherosclerosis. J Am Heart Assoc (2015) 4(4):e001403. 10.1161/JAHA.114.001403 25814626 PMC4579929

[37] Lo J Abbara S Shturman L Soni A Wei J Rocha-Filho JA Increased prevalence of subclinical coronary atherosclerosis detected by coronary computed tomography angiography in HIV-infected men. AIDS (2010) 24(2):243–53. 10.1097/QAD.0b013e328333ea9e PMC3154841 19996940

[38] Ruparelia N Godec J Lee R Chai JT Dall’Armellina E McAndrew D Acute myocardial infarction activates distinct inflammation and proliferation pathways in circulating monocytes, prior to recruitment, and identified through conserved transcriptional responses in mice and humans. Eur Heart J (2015) 36(29):1923–34. 10.1093/eurheartj/ehv195 PMC4571177 25982896

[39] Williams JW Zaitsev K Kim KW Ivanov S Saunders BT Schrank PR Limited proliferation capacity of aortic intima resident macrophages requires monocyte recruitment for atherosclerotic plaque progression. Nat Immunol (2020) 21(10):1194–204. 10.1038/s41590-020-0768-4 PMC7502558 32895539

[40] Kubota A Suto A Suzuki K Kobayashi Y Nakajima H Matrix metalloproteinase-12 produced by Ly6C(low) macrophages prolongs the survival after myocardial infarction by preventing neutrophil influx. J Mol Cell Cardiol (2019) 131:41–52. 10.1016/j.yjmcc.2019.04.007 31009606

[41] Carlin LM Stamatiades EG Auffray C Hanna RN Glover L Vizcay-Barrena G Nr4a1-dependent Ly6C(low) monocytes monitor endothelial cells and orchestrate their disposal. Cell (2013) 153(2):362–75. 10.1016/j.cell.2013.03.010 PMC3898614 23582326

[42] Clemente C Rius C Alonso-Herranz L Martin-Alonso M Pollan A Camafeita E MT4-MMP deficiency increases patrolling monocyte recruitment to early lesions and accelerates atherosclerosis. Nat Commun (2018) 9(1):910. 10.1038/s41467-018-03351-4 29500407 PMC5834547

[43] Tacke F Alvarez D Kaplan TJ Jakubzick C Spanbroek R Llodra J Monocyte subsets differentially employ CCR2, CCR5, and CX3CR1 to accumulate within atherosclerotic plaques. J Clin Invest (2007) 117(1):185–94. 10.1172/JCI28549 PMC1716202 17200718

[44] Combadiere C Potteaux S Rodero M Simon T Pezard A Esposito B Combined inhibition of CCL2, CX3CR1, and CCR5 abrogates Ly6C(hi) and Ly6C(lo) monocytosis and almost abolishes atherosclerosis in hypercholesterolemic mice. Circulation (2008) 117(13):1649–57. 10.1161/CIRCULATIONAHA.107.745091 18347211

[45] Lessard AJ LeBel M Egarnes B Prefontaine P Theriault P Droit A Triggering of NOD2 Receptor Converts Inflammatory Ly6C(high) into Ly6C(low) Monocytes with Patrolling Properties. Cell Rep (2017) 20(8):1830–43. 10.1016/j.celrep.2017.08.009 28834747

[46] Netea MG Joosten LA Latz E Mills KH Natoli G Stunnenberg HG Trained immunity: A program of innate immune memory in health and disease. Science (2016) 352(6284):aaf1098. 10.1126/science.aaf1098 27102489 PMC5087274

[47] Yao Y Jeyanathan M Haddadi S Barra NG Vaseghi-Shanjani M Damjanovic D Induction of Autonomous Memory Alveolar Macrophages Requires T Cell Help and Is Critical to Trained Immunity. Cell (2018) 175(6):1634–50. 10.1016/j.cell.2018.09.042 30433869

[48] Netea MG Dominguez-Andres J Barreiro LB Chavakis T Divangahi M Fuchs E Defining trained immunity and its role in health and disease. Nat Rev Immunol (2020) 20(6):375–88. 10.1038/s41577-020-0285-6 PMC7186935 32132681

[49] Kleinnijenhuis J Quintin J Preijers F Joosten LA Ifrim DC Saeed S Bacille Calmette-Guerin induces NOD2-dependent nonspecific protection from reinfection via epigenetic reprogramming of monocytes. Proc Natl Acad Sci U.S.A. (2012) 109(43):17537–42. 10.1073/pnas.1202870109 PMC3491454 22988082

[50] Bekkering S Quintin J Joosten LA van der Meer JW Netea MG Riksen NP Oxidized low-density lipoprotein induces long-term proinflammatory cytokine production and foam cell formation via epigenetic reprogramming of monocytes. Arterioscler Thromb Vasc Biol (2014) 34(8):1731–8. 10.1161/ATVBAHA.114.303887 24903093

[51] Zhong C Yang X Feng Y Yu J Trained Immunity: An Underlying Driver of Inflammatory Atherosclerosis. Front Immunol (2020) 11:284. 10.3389/fimmu.2020.00284 32153588 PMC7046758

