# Supplementary figures and images for: Mycobacterium bovis Bacille-Calmette-Guérin Infection Aggravates Atherosclerosis
Source: Front Immunol. 2020 Dec 18;11:607957. doi: 10.3389/fimmu.2020.607957 (PMC7775372; doi:10.3389/fimmu.2020.607957)

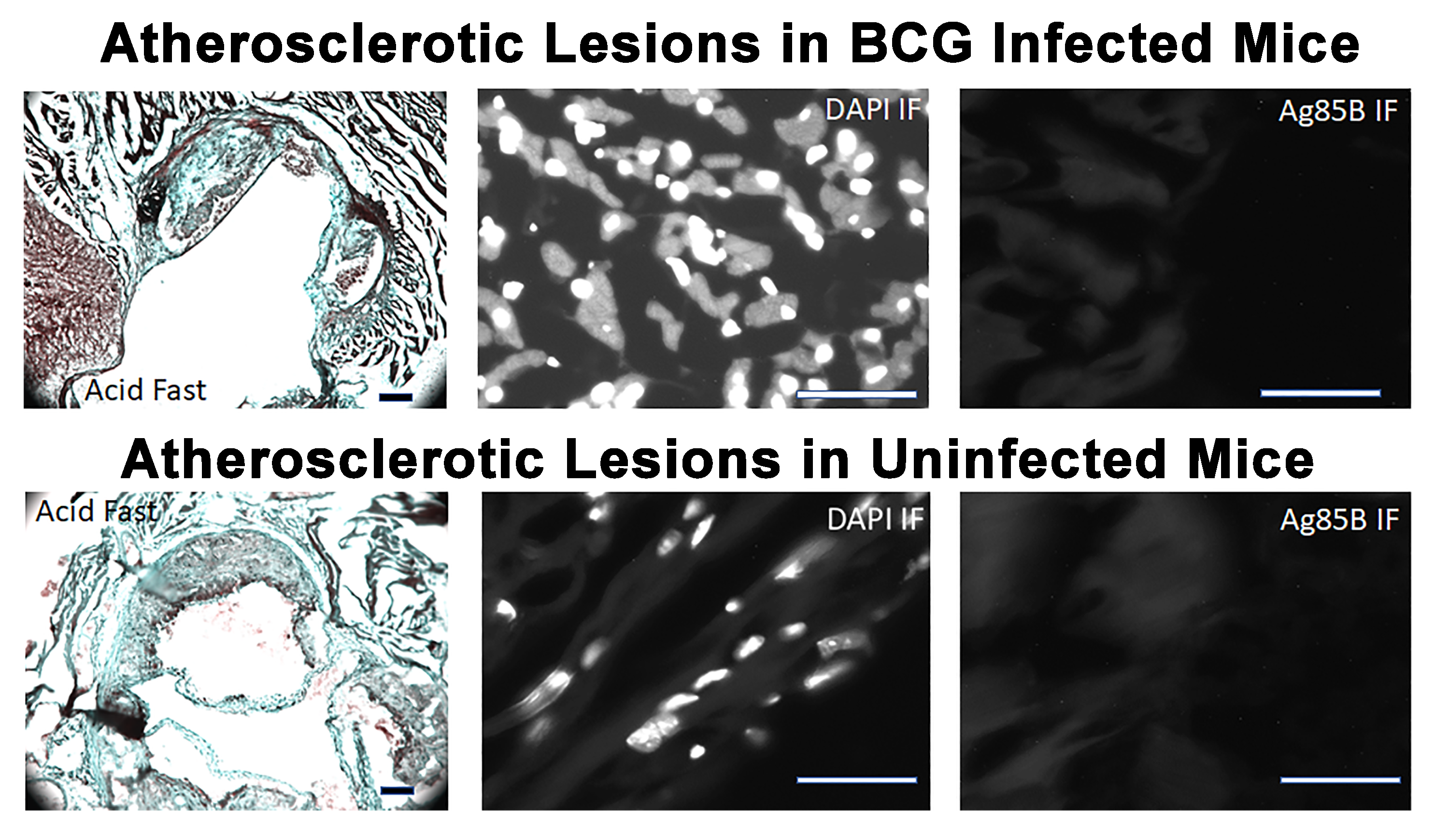

Supplement: Supplementary Figure 1 — Detection of mycobacteria in atherosclerotic lesions. Frozen sections of aortic roots isolated from M. bovis BCG-infected and uninfected Ldlr-/- mice were histologically stained with acid fast reagents by placing in Carbol-fuchsin solution (Sigma-Aldrich, catalog No. HT801) for 1 min followed by 2 min of counterstain with Malachite Green solution (Sigma-Aldrich, catalog No. HT802). The frozen sections were also subjected to immunofluorescence staining with the anti-Mycobacterium tuberculosis antibodies Ag85B (Abcam, catalog No. ab43019) with DAPI counterstain to identify the nucleus. The scale bars represent 100 µm. Note that no mycobacteria were detectable in the atherosclerotic lesions from either M. bovis BCG infected or uninfected mice. [file Image_1.tif]

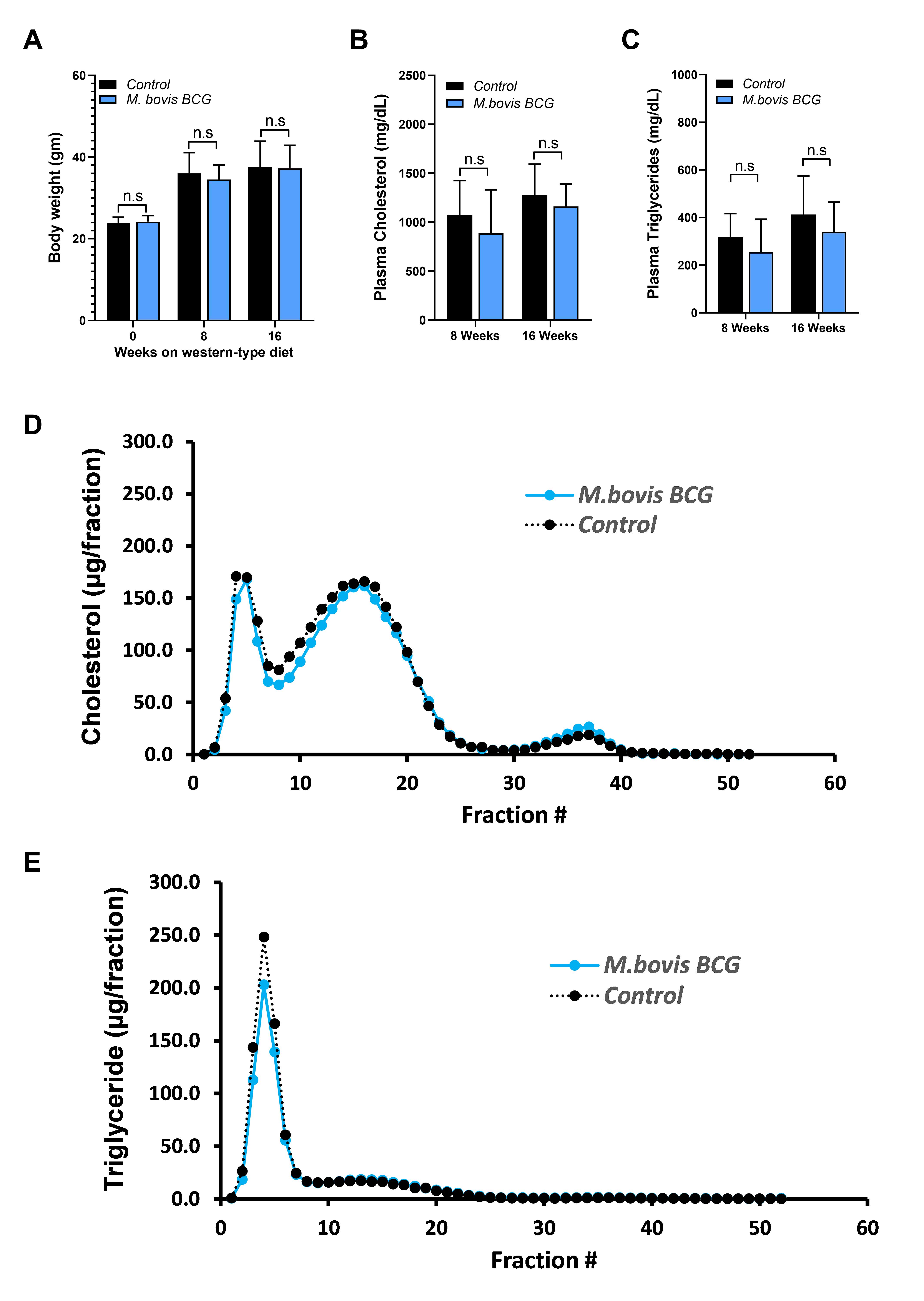

Supplement: Supplementary Figure 2 — M. bovis BCG infection does not induce significant changes in body weight or circulating lipids in Ldlr -/- mice. Ldlr-/- mice were inoculated with M. bovis BCG (0.3–3.0x10^6^ CFU) via the intranasal route. Mice were fed a western-type diet for 8 to 16 weeks. Age-matched uninfected Ldlr -/- mice fed with an identical diet served as controls. (A) Body weight at baseline, 8 weeks, and 16 weeks of western-type diet in M.bovis BCG infected (blue) and control mice (black). (B) Plasma cholesterol levels in mg/dL at weeks 8 and 16. (C) Plasma triglyceride levels in mg/dL at weeks 8 and 16. Data are means ± SD. n = 20 mice per group; pooled from 2 independent experiments. Significance was determined by Student’s t-test. n.s., non-significant (p > 0.05). Fast protein liquid chromatography (FPLC) profiles of total cholesterol (D) and tryglicerides (E) in M.bovis BCG infected and control mice at week 16. [file Image_2.jpeg]
